# Survey of knowledge and perception on the access to evidence-based practice and clinical practice change among maternal and infant health practitioners in South East Asia

**DOI:** 10.1186/1471-2393-8-34

**Published:** 2008-08-05

**Authors:** Ruth Martis, Jacqueline J Ho, Caroline A Crowther, The SEA-ORCHID Study Group

**Affiliations:** 1 Discipline of Obstetrics and Gynaecology, The University of Adelaide, Women's and Children's Hospital, King William Road, North Adelaide, South Australia, SA 5006, Australia; 2 Department of Paediatrics, Penang Medical College, Penang 10450, Malaysia

## Abstract

**Background:**

Evidence-based practice (EBP) can provide appropriate care for women and their babies; however implementation of EBP requires health professionals to have access to knowledge, the ability to interpret health care information and then strategies to apply care. The aim of this survey was to assess current knowledge of evidence-based practice, information seeking practices, perceptions and potential enablers and barriers to clinical practice change among maternal and infant health practitioners in South East Asia.

**Methods:**

Questionnaires about IT access for health information and evidence-based practice were administered during August to December 2005 to health care professionals working at the nine hospitals participating in the South East Asia Optimising Reproductive and Child Health in Developing countries (SEA-ORCHID) project in Indonesia, Malaysia, Thailand and The Philippines.

**Results:**

The survey was completed by 660 staff from six health professional groups. Overall, easy IT access for health care information was available to 46% of participants. However, over a fifth reported no IT access was available and over half of nurses and midwives never used IT health information. Evidence-based practice had been heard of by 58% but the majority did not understand the concept. The most frequent sites accessed were Google and PubMed. The Cochrane Library had been heard of by 47% of whom 51% had access although the majority did not use it or used it less than monthly. Only 27% had heard of the WHO Reproductive Health Library and 35% had been involved in a clinical practice change and were able to identify enablers and barriers to change. Only a third of participants had been actively involved in practice change with wide variation between the countries. Willingness to participate in professional development workshops on evidence-based practice was high.

**Conclusion:**

This survey has identified the need to improve IT access to health care information and health professionals' knowledge of evidence-based health care to assist in employing evidence base practice effectively.

## Background

Evidence-based practice (EBP) has been defined as the 'conscientious, judicious and explicit use of current best evidence available in making decisions about the care of individual patients', integrating individual clinical expertise, the needs and values of the individual patient and the best available research [1,2].

The understanding, accessing and implementing of EBP can provide appropriate and effective care in pregnancy, childbirth and the postnatal period for women and their babies [1]. However the wider application of EBP depends on the progress made in educating health professionals on accessing evidence-based research [3], evaluating and correctly interpreting research studies, the provision of quick, easy and free access to evidence [4] and an understanding on how to implement the findings into clinical practice [5].

Each year there are over half a million maternal deaths world-wide with 98% of these occurring in low and middle income countries. For women in Asia the lifetime risk of maternal death is one in 65 compared with one in 1,800 for women in high income countries [6]. Access to scientifically valid and up-to-date information is a prerequisite for providing evidenced-based care [4].

In the South East Asia Optimising Reproductive and Child Health In Developing countries (SEA-ORCHID) project, the three phases of the study included audit of the baseline rates and clinical care practice, an educational intervention to improve evidence-based care followed by an audit of change of rates and quality of clinical practice [7]. As part of the initial data collection in the four South East Asian (SEA) countries; Malaysia, Indonesia, Thailand and The Philippines, all clinical staff at the maternity, child health and newborn services of the nine participating hospitals were invited to participate in a survey of current knowledge, perceptions, activities, enablers and barriers to evidence-based practice.

## Methods

### Eligibility, sampling and time frame

The EBP survey was conducted among staff in the nine hospitals participating in the SEA-ORCHID project. These hospitals were situated in four South East Asian countries; Malaysia, Indonesia, Thailand and the Philippines. Of the nine hospitals, seven were tertiary referral institutions with regional referrals of women with a high risk pregnancy, one was a provincial institution and one was a district institution. The SEA-ORCHID project settings and methods have been published elsewhere [[7] and see Additional file 1].

All maternal and child health staff working on roster at the nine participating hospitals across South-East Asia were invited to participate in the EBP survey during August to December 2005. The EBP survey was approved by all relevant Ethics Committees at the SEA-ORCHID participating sites.

The SEA-ORCHID project was interested in obtaining information about the knowledge and access of on-line evidence-based information. The Cochrane Library [8] is one such on-line resource, reliable for obtaining evidence-based clinical information and up-to-date systematic reviews in health care. The World Health Organization (WHO) Reproductive Health Library (RHL) [9] is another on-line evidence-based resource. It aims to put the best available evidence into a practical context so that it can be used to improve health outcomes. RHL started in 1997 and is prepared by an editorial team based in the WHO department of reproductive health and research and other international partner institutions. The audit sought detail on the involvement of participants with clinical practice change and possible barrier and enabler identification in their clinical area.

At each site the distribution of the questionnaires was managed by the local SEA-ORCHID study researcher. Instructions on how to administer the survey and how the data was to be entered on-line were emailed to all local study researchers, who instructed the field workers as to the correct on-line data entry. The instructions were also posted on-line for easy access on the SEA-ORCHID web site (please see Availability & requirements for more information) that was protected by a password for access. Zoomerang software (please see Availability & requirements for more information) was used for on-line data entry and entered data was converted into Excel for data checking and analyses.

### The survey questionnaire

The survey consisted of 39 questions and was developed and piloted by one of the SEA-ORCHID clinical educators (RM) with feedback from SEA and Australian investigators [see Additional file 2]. The pilot data were not included in the analyses. The survey development was assisted by having access to and permission for use of a WHO RHL survey tool [10].

Most survey questions needed to be answered by writing a number in a box next to listed responses, 1 indicating no, 2 indicating yes and 3 indicating maybe. Most questions gave the opportunity to specify additional information. The survey comprised seven sections. The first section collected demographic data and IT technology available at the participant's work place. The second assessed the health information sources used by participants, how often they consulted such sources and how much time was spent reading health information. EBP beliefs were addressed in section three as well as whether the participant was an author of a systematic review. Section four and five sought information about participant's knowledge of the Reproductive Health Library (RHL) [9] and The Cochrane Library [8]. Section six explored participant's involvement and experiences with clinical practice change including barrier identification and how to overcome these. Finally participants identified workshops they would like to attend for enhancing their understanding of EBP and what would prevent them from attending such workshops.

### Statistical analysis

Data analysis was conducted using pivotTables in Microsoft Office Excel 2003 calculating frequency and corresponding percentages to describe the responses to the survey questions for all participating health professionals and combining the data for all four SEA countries. Two response questions were presented as a list and frequency of occurrence rather than linked to health professionals or given as percentages.

### Ethical approval

The SEA-ORCHID project was approved by the local ethics committees of each hospital and by the ethics committee of the University of Sydney, the administering institution in Australia.

## Results

### Demographics

A total of 660 staff across the four SEA-ORCHID countries were surveyed. Professions represented included 80 obstetrics and gynaecology (O&G) specialists, four neonatologists, 36 paediatricians, 158 postgraduate medical trainees, 263 nurses, and 119 midwives (nurses with midwifery qualifications included).

The participants were identified as 564 (85%) female and 96 (15%) male across the four participating SEA countries (Table 1). The female gender dominated for all professions surveyed, with nurses represented by 263 (100%) females, closely followed by midwives with 118 (99%). The age range varied among the professions with an overall mean age of 36 ± 9 years. A mean of 9 ± 8 years practicing in participants' respective profession was represented (Table 1).

**Table 1:** Demographics: gender, age range and years practicing in profession across all four SEA countries (n = 660)

**Profession**	**Total**	**Gender ** **n (%)**		**Age**	**Years practicing**
		
		**Male**	**Female**	**Mean ± S.D.**	**Mean ± S.D.**
O & G specialist	80	38 (48)	42 (53)	39 ± 10	9 ± 9
Neonatologist	4	1 (25)	3 (75)	41 ± 5	11 ± 6
Paediatrician	36	14 (39)	22 (61)	39 ± 8	9 ± 8
Postgraduate medical trainee^a^	158	42 (27)	116 (73)	29 ± 3	3 ± 2
Nurse	263	0 (0)	263(100)	35 ± 8	10 ± 7
Midwife^b^	119	1 (1)	118 (99)	42 ± 8	13 ± 9

**Total**	**660**	**96 (15)**	**564 (85)**	**36 ± 9**	**9 ± 8**

^a^Identified as Resident or RMO in Thailand, Indonesia, The Philippines and Medical Officer in Malaysia

^b^Nurse with midwifery training included

All figures are rounded to the nearest whole number

### In-service training

Of the overall participants, 368 (56%) commented that the type of in-service training usually offered at their institution was of technical or professional nature, rather than managerial or administrative. No in-service training was indicated by 66 (10%) and 13 (2%) did not know if training was offered. However, 201 (30%) indicated that both types (managerial or administrative and technical or professional) of in-service training were offered at their institution.

### Access to IT services

In answer to the question of easy access to a computer at the workplace, 301 participants across all four SEA countries (46%) responded that they had access with broadband internet connection while 139 (21%) indicated that they had no access. However there were great variations between the countries. In Indonesia, 63 (50%) of all participants reported no access to computers and of the participants that did, 26 (21%) could not access the internet through their computer. Thailand had the highest reported access where 127 (84%) participants had easy access to a computer at work with broadband connection (Table 2).

**Table 2:** Access to computers and IT services by participating SEA countries and by profession (n = 660)

	**No access ** **n (%)**	**Yes, without internet connection ** **n (%)**	**Yes, with phone internet connection ** **n (%)**	*Yes, with broadband internet connection **n (%)**
**Country**				

Malaysia	42 (15)	83 (30)	41 (15)	110 (40)
Indonesia	63 (50)	26 (21)	5 (4)	32 (25)
Thailand	6 (4)	8 (5)	10 (7)	127 (84)
Philippines	28 (26)	15 (14)	32 (30)	32 (30)

**Total**	**139 (21)**	**132 (20)**	**88 (13)**	**301 (46)**

**Profession**				

O&G Specialist	5 (6)	9 (11)	10 (13)	56 (70)
Neonatologist	0 (0)	0 (0)	0 (0)	4 (100)
Paediatrician	2 (6)	0 (0)	5 (14)	29 (81)
Postgraduate medical trainee^a^	19 (12)	22 (14)	43 (27)	74 (47)
Nurse	76 (29)	58 (22)	23 (9)	106 (40)
Midwife^b^	37 (31)	43 (36)	7 (6)	32 (27)

**Total**	**139 (21)**	**132 (20)**	**88 (13)**	**301 (46)**

*Identified as easy access

^a^Identified as Resident or RMO in Thailand, Indonesia and The Philippines and Medical Officer in Malaysia

^b^Nurse with midwifery training included

All figures are rounded to the nearest whole number

Of all the SEA participants with no easy access to a computer at their workplace, 114 (50%) indicated that the greatest difficulty was a limited supply of computers. The 'other' option was chosen by 14 (6%) participants where 7 (50%) described that the difficulty they had was due to their limited knowledge of how to use the computer or the available software on the computer.

### Health Information and Resources

Participants across all four SEA countries indicated that the reason for consulting health information sources 'frequently' were for patient care (377, 57%), for teaching (216, 33%), for personal study (219, 33%) and for research (179, 27%).

Resources 'frequently' used for health information were text books (359, 54%), colleagues (212, 32%), journals (157, 24%) and resources from pharmaceutical companies (30, 5%). Resources 'sometimes' used were resources from pharmaceutical companies (438, 66%), journals (380, 57%), colleagues (347, 52%) and textbooks (278, 42%).

The internet was used as a resource for gaining health information by 409 participants (62%), who listed 46 websites, with the most popular website being  129 (20%), closely followed by  69 (10%), then  62 (9%) and  50 (8%) (Table 3). Across SEA, nurses were the least likely to use the internet for health information with 136 (52%) indicating 'never', followed by nurse-midwives and postgraduate medical trainees. When asked how much time participants actually spent reading health information on an average weekly basis, 180 (27%) indicated less than one hour, 290 (44%) 1–2 hours and 195 (29%) indicated 3 or more hours a week.

**Table 3:** The four web sites used mostly for health information access by profession across SEA (n = 660)

*Profession	**Google ** **n (%)**	**PubMednl ** **n (%)**	**PubMed Central ** **n (%)**	**Yahoo ** **n (%)**
O&G Specialist	18 (23)	17 (21)	14 (18)	11 (14)
Neonatologist	3 (75)	4(100)	4(100)	0 (0)
Paediatrician	14 (39)	11 (31)	11 (31)	4 (11)
Postgraduate medical trainee^a^	32 (20)	33 (21)	30 (19)	13 (8)
Nurse	54 (21)	3 (1)	2 (1)	18 (7)
Midwife^b^	8 (7)	1 (1)	1 (1)	4 (3)

**Total**	**129 (20)**	**69 (10)**	**62 (9)**	**50 (8)**

*Please note that some professions might have indicated more than one option

^a^Identified as Resident or RMO in Thailand, Indonesia and The Philippines and Medical Officer in Malaysia

^b^Nurse with midwifery training included

All figures are rounded to the nearest whole number

### Evidence-Based Practice

All participants were asked if they had ever heard about evidence-based practice, evidence-based medicine or evidence-based care. Of the total number of survey participants, 385 (58%) had heard about the concept. This result differed considerably between countries and the health professions, with the data from individual countries showing that 114 (75%) of Thailand's clinicians had heard about EBP, 71 (66%) from the Philippines, 142 (51%) from Malaysia and 60 (48%) from Indonesia.

Among SEA health professionals the different groups who identified that they had heard about EBP were 94 (36%) nurses, 48 (40%) midwives, 134 (85%) postgraduate medical trainees, 71 (90%) O&G specialists, 34 (97%) paediatricians and 4 (100%) neonatologists (Table 4). Of the participants that had heard about EBP, 291 (76%) opted to write down their personal definition. The answers varied considerably between participants; however the majority of responses indicated a lack of understanding of the EBP concept identifying it as clinical practice guidelines or something to do with research.

**Table 4:** Heard about EBP, The Reproductive Health Library and The Cochrane Library by profession across SEA (n = 660)

**Profession**	**Reproductive Health Library**	**The Cochrane Library**	**EBP**
	**n (%)**	**n (%)**	**n (%)**
O&G Specialist	46 (82)	71 (89)	71 (90)
Neonatologist	4(100)	4(100)	4(100)
Paediatrician	9 (35)	32 (89)	34 (97)
Postgraduate medical trainee^a^	69 (83)	121 (77)	134 (85)
Nurse	44 (32)	63 (24)	94 (36)
Midwife^b^	5 (4)	16 (13)	48 (40)

**Total**	**177 (27)**	**307 (47)**	**385 (58)**

^a^Identified as Resident or RMO in Thailand, Indonesia and The Philippines and Medical Officer in Malaysia

^b^Nurse with midwifery training included

All figures are rounded to the nearest whole number

### The Cochrane Library

In relation to having heard about The Cochrane Library, 307 (47%) participants indicated that they had (Table 4) with 156 (51%) of these having access to it. This is obviously related to on-line computer access and whether a subscription had been paid. However, of the participants that had access to The Cochrane Library, 28 (18%) indicated that they never used it, 30 (19%) used it once a year, 65 (42%) used it once a month, 28 (18%) used it once a week and 12 (8%) used it more than once a week. This was a consistent result across all four SEA countries. A question relating to whether participants found The Cochrane Library helpful was answered by 140 participants with 83 (59%) indicating they found it a helpful tool. The usefulness related to accessing systematic reviews (29, 47%) and to retrieving information for clinical practice guideline development (11, 18%). Of all the survey participants 311 (70%) indicated they had not attended a Cochrane Library workshop and 571 (81%) expressed interest in attending such a workshop. This was consistent across all four countries with a range of 79–85%.

### The Reproductive Health Library (RHL)

Within the survey participants 177 (27%) had heard about the RHL and of these 83 (47%) had access to it (Table 4). Knowing about the RHL varied considerably between countries. In Indonesia, 6 (5%) health professionals had heard about this on-line resource, in the Philippines 23 (21%), in Malaysia 25 (9%) and in Thailand 46 (30%). RHL is a free resource to low and middle income countries.

Only 70 participants answered the question on whether RHL tool is helpful in their practices of which 57% answered that it is useful but 43% answered it is useful sometimes. Both groups indicated the most useful section in the RHL were the systematic reviews (42, 60%). Of these 70 participants, 28 (40%) had attended a RHL workshop, mainly in Thailand. No participants in the Philippines or Malaysia and only two from Indonesia had attended a RHL workshop. Overall 572 (87%) of health professionals across SEA indicated an interest in attending a RHL workshop. This was consistent in all four countries with a range of 80–89%.

### Clinical Practice Change

Of the survey participant 230 (35%) responded as having been involved in changing an established clinical practice. These results varied between the four SEA countries however with participant from Thailand indicating 112 (74%) had been involved, 36 (29%) in Indonesia, 72 (26%) in Malaysia and 10 (9%) in The Philippines. These participants were then asked to identify who initiated this change with 75 (33%) identifying that the change was initiated by the Head of the Department, followed by the respondent themselves (54, 23%), senior staff (46, 20%) and colleagues (37, 16%) (Table 5).

**Table 5:** Who initiated changing an established clinical practice by profession across four SEA countries (n = 660)

**Profession**	**Team**	**You**	**Colleague**	**Senior Staff**	**Head of Department or Management **	**Don't know **	**Total**
	**n (%)**	**n (%)**	**n (%)**	**n (%)**	**n (%)**	**n (%)**	**n (%)**
O&G Specialist	3 (8)	14 (36)	8(21)	6(15)	7 (18)	1 (3)	80 (12)
Neonatologist	1(25)	3 (75)	0 (0)	0 (0)	0 (0)	0 (0)	4 (1)
Paediatrician	1 (4)	14 (58)	1 (4)	1 (4)	7 (29)	0 (0)	36 (5)
Postgraduate medical trainee^a^	1 (4)	2 (7)	1 (4)	13 (46)	10 (36)	1 (4)	158 (24)
Nurse	8 (7)	20 (18)	26 (23)	19 (17)	38 (34)	1 (1)	263 (40)
Midwife^b^	0 (0)	0 (0)	1 (4)	7 (28)	13 (52)	4 (16)	119 (18)

**Total**	**14 (6)**	**54 (23)**	**37 (16)**	**46 (20)**	**75 (32)**	**7 (3)**	**660**

^a^Identified as Resident or RMO in Thailand, Indonesia and The Philippines and Medical Officer in Malaysia

^b^Nurse with midwifery training included

All figures are rounded to the nearest whole number

Participants were asked about their understanding of why the change was made. Across SEA, 125 (54%) participants responded that the change was initiated because of new evidence, 70 (30%) identified the change was made because new health technology was made available, 15 (7%) indicated the change was made due to a new pharmaceutical drug and 16 (7%) did not know the reason for the initiated clinical practice change. Minor and major resistance to changing clinical practice was reported by 158 (69%) participants with the major reason being no discussion at implementation stage (67, 42%). This was followed by identifying additional resistance to change as no or little consultation (39, 25%), difficulty in accessing new clinical guidelines (28, 18%) and the observation that people did not like change or held on to their different clinical opinions and beliefs (15, 9%).

Participants were invited to identify possible enablers to overcome the barriers that were identified in the previous question. The major recommended action was discussion groups within professional groups (76, 48%) from inception right through to implementation. Easy access to clinical practice guidelines were identified by 31 (20%) participants and multidisciplinary workshops by 30 (19%). Overall, a local language was not identified as needed for enabling change or overcoming barriers. However, 16 (10%) of Thailand's participants indicated that translation of clinical practice guidelines would be useful.

### Professional Development Indication

Participants were invited to indicate their interest in attending workshops on evidence-based health care and to provide suggestions for workshop topics as this could assist their professional development. Across all four SEA countries the majority of health professionals surveyed were interested in attending such workshops with a range from 550 (83%) to 578 (88%) (Table 6). Additional topics for workshops were suggested by 9 participants, mainly related to computing skills and internet searching.

**Table 6:** Workshops interested in attending by health professionals across four SEA countries (n = 660)

**Profession**	**RHL**	**Cochrane**	**EBP**	**CPG**	**Use of evidence **
	**n (%)**	**n (%)**	**n (%)**	**n (%)**	**n (%)**
O&G Specialist	74 (93)	71 (88)	72 (90)	69 (88)	72 (90)
Neonatologist	3 (75)	3 (75)	3 (75)	3 (75)	3 (75)
Paediatrician	25 (69)	30 (83)	31 (86)	30 (83)	31 (86)
Postgraduate medical trainee^a^	141 (89)	135 (85)	137 (87)	138 (87)	137 (87)
Nurse	228 (87)	211 (80)	210 (80)	227 (86)	229 (87)
Midwife^b^	97 (82)	97 (82)	95 (80)	100 (84)	102 (87)

**Total**	**568 (86)**	**547 (83)**	**548 (83)**	**567 (86)**	**574 (87)**

^a^Identified as Resident or RMO in Thailand, Indonesia and The Philippines and Medical Officer in Malaysiam

^b^Nurse with midwifery training included

All figures are rounded to the nearest whole number

It is of interest to the SEA-ORCHID project to understand if there are reasons that would prevent participants from attending the workshops for professional development. The survey data showed that 211 (32%) participants had no concerns, 353 (53%) had concerns and 101 (15%) indicated that maybe there were concerns. Of those that identified concerns, 271 (41%) indicated they needed financial support but none was available, 260 (39%) felt they were too busy with their clinical workload and 182 (28%) indicated language as a barrier. Other barriers were indicated by 14 people with the two main reasons being difficulty in obtaining permission from Head of Department and difficulty in changing shifts.

## Discussion

Our findings provide a useful foundation from which to plan future interventions and directions for EBP among maternal and child health professionals in SEA. The survey adds to the hospital data available from South East Asian countries on EBP knowledge amongst health professionals. The sample size of the study was limited to sites participating in the SEA-ORCHID project that may not be representative of all hospitals within each of the countries. Investigators at the hospitals that were chosen for the study were interested in EBP, so it is likely that these sites had more exposure to EBP. The survey results, therefore, would be likely to over-estimate EBP, knowledge and clinical change in reference to South East Asia as a whole.

### IT Access

Less than half of participants had easy access to computers with broadband internet connections although an additional 13% were able to access the internet via a dial-in (telephone) connection. Across the participating SEA countries dial-in can be difficult to access on-line information, as the phone connections experience frequent interruptions. IT access varied widely between participating countries. Easy internet access is fundamental to inform EBP and to access EBP resources. It is an important tool to use for up-to-date information and latest research findings. Easy on-line access ensures busy clinicians are able to inform their practice through accessing peer-reviewed journals and updated research summary evidence such as those available through The Cochrane Library and The RHL [11].

Major barriers to accessing knowledge include difficulties with internet access, high costs of mailing and subscriptions [12]. These difficulties are greater for health workers outside research settings involved mainly in routine care [13].

Our data shows the more senior the health professional the more likely it was for the IT access to be easy. For all the SEA participants that indicated they had no easy access to a computer at their workplace, half indicated that the greatest difficulty was a limited supply of computers. For some participants the difficulty they had was due to their limited knowledge of how to use the computer. This indicates an area where resources, training and assistance need to be allocated and planned in the workplace. Similar indications were found in an EBP survey among American physical therapists [14] where physical therapists that had easy access to online resources were likely to perform data searches more frequently and tended to read more articles [14].

### Health Information, Resources and EBP understanding

EBP resources are growing fast. For health professionals to access these not only do they need to be able to have easy online access but also a knowledge about databases and how to search, as well as critical appraisal skills to interpret the information. Survey participants were asked how often and how much time they would spend consulting health information resources. Overall, the participants indicated that they consult any health information primarily for patient care. Textbooks were almost universally used with other highly used sources to inform clinical practice including colleagues, journals and information from pharmaceutical companies. Text books however, are quickly outdated, colleagues, while well experienced staff may be more opinion-based in their care rather then evidence-based and information from pharmaceutical companies may be biased towards their own research and products.

The internet was used by two thirds of participants with the most popular URL being , which is not necessarily an evidenced-based database. Nurses and nurse-midwives were the least likely to use the internet for health information, followed by postgraduate medical trainees. All this information is useful in shaping future education programs within the SEA-ORCHID hospitals.

Wider application of EBP in clinical practice will largely depend on the progress made in the area of educating health professionals in accessing peer reviewed evidence and the provision of quick and easy access to evidence, in particular if the evidence is formatted into clinical summaries [11,15]. The lack of awareness of databases of pre-appraised evidence and the lack of access to these databases are recognized as barriers to practicing EBP [16].

Although half of the survey participants were aware of The Cochrane Library and one third of RHL, less than one-third actually used them on a regular basis. However the majority of participants were unaware of the existence of The Cochrane Library or RHL.

The majority of participants indicated that they read health information for 1–2 hours a week. This level of attention to reading literature may not necessarily mean substandard care. Experienced health professionals who provide care frequently on a daily basis for a similar patient population may not need to refer to the literature as often. Three quarters of our participants consulted journals for informing their clinical practice. Most peer reviewed journals are published monthly or less frequent and maybe this level of reading on a weekly basis could be adequate. This of course relates to the actual material read and whether it is evidence-based [14,2].

Only half of our participants had heard about the concept of EBP. This significant finding highlights the importance of readily accessible resources and opportunities for professional development workshops on EBP for busy health professionals.

### Involvement in and Barrier and Enabler Identification for Clinical Practice Change

Over one third of participants had been involved in changing an established clinical practice. Relatively few participants did not know the reason for the change indicating good communication when implementing clinical practice change across the surveyed SEA sites. However, many participants reported resistance of differing degrees, with the main reason being no discussion at the implementation stage. Eccles and Grimshaw [17] indicated there are 'no magic bullets' but involving people to change clinical behaviour rather than 'telling people to change' is an important factor for success.

Barrier analysis can assist people with the planning and implementation of clinical practice change. Efforts can then be focused on interventions tailored to address specific barriers and identified enablers [18] and is a recommended practice. In the literature it has been noted that barriers to change can occur across different levels of health care and analysis therefore needs to include policy makers and other stake holders, such as patients [5,19,20].

Enablers for clinical practice change were identified by participants. Overwhelmingly participants stated that discussion groups within professional groups from inception right through implementation would be the most effective way of ensuring sustainable clinical practice change. Easy access to evidence-based clinical practice guidelines was also identified. It is known that the 'barriers' reported as preventing implementation are less important than the context and underlying social relations that have given cause to them [21]. While it was difficult to assess from our survey what the context of clinical practice change was and what the underlying social relations were, participants indicated that they wanted to have discussions within their individual professional groups rather than multidisciplinary groups. This may be because of important underlying social relations.

### Identified Professional Development Needs and Identified Challenges

Participants were able to identify their professional development needs and how these might be met. The majority requested training in the use of RHL and The Cochrane Library, Clinical Practice Guideline development and how to implement evidence into clinical practice. Other workshop suggestions were computer skill courses and how to search the internet. This was fairly consistent across all countries and among all health professional groups surveyed. Difficulty in attending workshops or training programmes, such as due to lack of available financial support and busy clinical workload would need to be overcome. These are echoed in the literature [22,23] and are always a challenge to overcome.

## Conclusion

In conclusion, this EBP survey across four SEA countries indicates that a concerted effort by those promoting EBP are needed to ensure all clinicians, including nurses, midwives and other allied health professionals, are able to practice EBP. This will raise their awareness of evidence-based clinical practice guidelines and access to EBP resources on-line. In particular the lack of awareness of databases of pre-appraised evidence and the lack of access to these databases highlights some of the issues of practicing EBP effectively.

## Competing interests

The authors declare that they have no competing interests.

## Availability & requirements

SEA-ORCHID web site: 

Zoomerang software: 

Google: 

PubMed NL: 

PubMed Central: 

Yahoo: 

## Authors' contributions

All authors contributed to the study design, interpretation of the data and preparation of the drafts of the manuscript. In addition all authors coordinated the trial and the collection of data. All authors read and approved the final manuscript.

## Funding

The SEA-ORCHID study is jointly funded by an International Collaborative Research Grant from the National Health and Medical Research Council of Australia (No. 307703) and Wellcome Trust, United Kingdom (071672/Z/03/Z). All authors were funded individually by their respective university/institution for the preparation of the project proposal.

## Pre-publication history

The pre-publication history for this paper can be accessed here:



## Supplementary Material

Additional file 1 Sea-orchid protocol. Optimizing reproductive and child health outcomes by building evidence-based research and practice in South East Asia (SEA-ORCHID): study protocol Click here for file

Additional file 2 Staff survey. Evidence-Based Practice Survey Click here for file
