# Supplementary material for: Survey of knowledge and perception on the access to evidence-based practice and clinical practice change among maternal and infant health practitioners in South East Asia
Source: BMC Pregnancy Childbirth. 2008 Aug 5;8:34. doi: 10.1186/1471-2393-8-34 (PMC2533287; doi:10.1186/1471-2393-8-34)
Supplement: Additional file 2 — Staff survey. Evidence-Based Practice Survey [file 1471-2393-8-34-S2.pdf]

Evidence-Based Practice Survey (STAFF only) TH (A)

SEA-ORCHID

- Please complete this form if you are a clinical staff member at the maternity, child health or newborn service.
- Please answer all questions with a **number in the boxes provided** and/or write additional comments where required.

General Information

1. Today's date ___/___/2005
2. Age at last birthday
3. Gender
   1 = male 2 = female
4. Please state which hospital you are from
   ____
5. Profession
   1 = Specialist in O & G
   2 = Neonatologist
   3 = Paediatrician
   4 = Resident (RMO)
   5 = Nurse
   6 = Midwife
   7 = Other, please specify
   ____
6. Years practising in the profession above (in Q.5)
7. Do you belong to a professional organisation(s)?
   1 = no
   2 = yes, please specify
   ____
   ____
   ____
8. What type of in-service training is usually offered in your workplace?
   1 = none
   2 = technical/professional
   3 = administrative/managerial
   4 = both of the above
   5 = do not know
   6 = other, please specify
   ____
9. Do you have easy access to a computer at your workplace?
   1 = no
   2 = yes, without internet connection
   3 = yes, with phone internet connection
   4 = yes, with broadband internet connection
10. If you answered **no** to question 9, what **greatest** difficulty do you have in accessing a computer with internet connection?
    1 = located too far from my workplace
    2 = booking required
    3 = unreliable internet connection
    4 = limited supply of computers available
    5 = computers always in use
    6 = have to use my own computer at home
    7 = other, please specify
    ____

Health Information Needs

11. What are generally the reasons you consult health information sources? **Please respond with 1, 2, 3, as below**
    1 = never 2 = sometimes 3 = frequently
    a) patient care
    b) teaching
    c) research
    d) personal study
    e) other reasons, please specify
    ____

Survey record no.____________/____________
(for office use only, don't fill in)

12. How often do you use the following resources? **Please respond with 1, 2 or 3, as below**
    1 = never 2 = sometimes 3 = frequently
    a) textbooks
    b) journals
    c) resources from pharmaceutical companies
    d) colleagues
    e) conferences
    f) staff meetings
    g) internet, please specify which websites you find most helpful
    ____
    ____
    h) other, please specify
    ____
13. How much time do you spend reading work-related literature in an average week?
    1 = less than one hour
    2 = 1-2 hours
    3 = 3 or more hours
14. In the past year, have you participated in any of the following professional activities related to your area of work, either as a participant or presenter? **Please respond with 1 or 2**
    1 = no 2 = yes
    a) conference/congress
    b) training workshop
    c) seminar
    d) case study presentation
    c) other, please specify
    ____

Evidence-Based Practice

15. Have you heard about any of the following: evidence-based practice, evidence-based care or evidence-based medicine?
    1 = no 2 = yes
    If you answered yes, what do you understand by evidence-based practice?
    ____
    ____
    ____
    ____
16. Have you ever attended a course or workshop on evidence-based practice/care/medicine?
    1 = no 2 = yes
17. Are you or have you been an author on a systematic review?
    1 = no 2 = yes, state what topic(s) you reviewed
    ____
    ____
    ____

Knowledge About The Cochrane Library

18. Have you heard about The Cochrane Library?
    1 = yes 2 = no, **please go to question 25**
19. Do you have access to The Cochrane Library?
    1 = yes 2 = don't know
    3 = no, **please go to question 24**

Evidence-Based Practice Survey (STAFF only) cont. TH (A)

SEA-ORCHID

20. If you have access, how often do you use The Cochrane Library?
    1 = never, **please go to question 24**
    2 = once a year
    3 = once a month
    4 = once a week
    5 = more than once a week

21. Where do you **most often** access The Cochrane Library?
    1 = at home
    2 = on your ward
    3 = within your department
    4 = the hospital library
    5 = other hospital wards
    6 = other hospital departments
    7 = other, please specify

    ____

22. Do you find The Cochrane Library a helpful tool for your clinical practice?
    1 = yes   2 = sometimes
    3 = no, **please go to question 24**

23. If you answered **yes**, please comment on what in particular you find useful.

    ____

    ____

24. Have you ever attended a Cochrane Library workshop?
    1 = yes   2 = no

Knowledge About WHO RHL

25. Have you heard about the WHO Reproductive Health Library (RHL)?
    1 = yes   2 = no, **please go to question 32**

26. Do you have access to RHL?
    1 = yes   2 = do not know
    3 = no, **please go to question 31**

27. If you have access to RHL, how often do you use it?
    1 = never, **please go to question 31**
    2 = once a year
    3 = once a month
    4 = once a week
    5 = more than once a week

28. Where do you **most often** access RHL?
    1 = at home
    2 = on your ward
    3 = within your department
    4 = the hospital library
    5 = other hospital wards
    6 = other hospital departments
    7 = other, please specify

    ____

29. Do you find RHL a helpful tool for your clinical practice?
    1 = yes   2 = sometimes
    3 = no, **please go to question 31**

30. If you answered **yes** or **sometimes**, what in particular do you find useful?
    1 = video clips/practical aspects
    2 = commentaries
    3 = systematic reviews
    4 = internet links
    5 = other, please specify

    ____

31. Have you ever attended a RHL workshop?
    1 = yes   2 = no

Clinical Practice Change

32. Have you ever been involved in changing an established clinical practice?
    1 = yes   2 = no, **please go to question 38**

33. If you answered **yes**, who initiated the change?
    1 = you
    2 = a colleague
    3 = senior staff
    4 = head of department/management
    5 = do not know
    6 = other, please specify

    ____

34. In your opinion, why was the change made?
    1 = new evidence
    2 = new drug available
    3 = new procedure/health technology available
    4 = legal implications
    5 = do not know
    6 = other, please specify

    ____

35. Was there any resistance to changing clinical practice?
    1 = no, **please go to question 38**
    2 = yes, minor resistance
    3 = yes, major resistance

36. If you answered **yes**, in your opinion, what was the **major reason** for the resistance?
    1 = no or little consultation prior to change
    2 = no discussions provided at implementation stage for staff
    3 = language difficulties
    4 = difficulty accessing new clinical guidelines
    5 = other, please specify

    ____

    ____

37. In your opinion, what would be the best course of action to over-come the main identified reason for resistance?
    1 = translation of guidelines
    2 = multidisciplinary workshops
    3 = discussion groups within professional groups
    4 = provide easy access to guidelines
    5 = other, please specify

    ____

    ____

Workshop Attendance

38. Would you be interested in attending any of the workshops listed below? **Please respond with 1, 2 or 3, as below**

    1 = yes   2 = no   3 = maybe

    a) How to use the RHL more effectively?
    b) How to access information from the Cochrane Library?
    c) EBP workshop including critical appraisal and under-standing systematic reviews
    d) Developing clinical practice guidelines
    e) Implementing evidence into clinical practice
    f) Other workshop suggestion(s), please specify

    ____

    ____

39. If you answered **yes**, what would prevent you from attending such a workshop? **Please respond with 1, 2 or 3, as below**

    1 = yes   2 = no   3 = maybe

    a) = nothing
    b) = too busy
    c) = language barrier
    d) = no financial support
    e) = other, please specify

    ____

Thank you for your time and participation with this survey. Please return form to:
